# Dosing practices of caffeine therapy for apnoea of prematurity: a retrospective single-centre observational study

**DOI:** 10.1136/bmjpo-2025-004301

**Published:** 2026-03-03

**Authors:** Odunayo Adebukola Temitope Fatunla, Coen S Zandvoort, Shellie Robinson, Eleri Adams, Caroline Hartley

**Affiliations:** 1 Department of Paediatrics, University of Oxford, Oxford, UK; 2 Newborn Care Unit, John Radcliffe Hospital, Oxford, UK

**Keywords:** Neonatology, Intensive Care Units, Neonatal, Therapeutics, Infant

## Abstract

**Objective:**

To evaluate caffeine prescribing practices in a tertiary neonatal unit, focusing on initiation, dose adjustment, discontinuation and recommencement, and to assess associations with gestational age and respiratory support.

**Design:**

Retrospective observational study.

**Setting:**

Neonatal unit, John Radcliffe Hospital, Oxford, United Kingdom.

**Patients:**

Preterm infants born ≤32 weeks gestation and admitted between 1 February 2022 and 31 October 2023. Data extracted from paper patient records included daily caffeine dosing, initiation, discontinuation, recommencement, coadministration with doxapram, demographics and duration of respiratory support. Associations between caffeine administration and clinical factors such as gestational age were assessed using regression.

**Results:**

168 admissions were analysed from 163 infants. Caffeine was typically initiated with a loading dose of 20 mg/kg, and maintenance doses ranged from 5 mg/kg/day to 25 mg/kg/day. There were 1–8 dose adjustments per admission. Doxapram was administered to 19 infants. Caffeine was discontinued at a median (IQR) postmenstrual age of 34.0 (33.9–34.7) weeks and was recommenced in four infants. Gestational age at birth was negatively correlated with postmenstrual age at discontinuation (r(CI) –0.33 (–0.51 to –0.12), p=0.0029; R²=0.11) and infants born at lower gestational ages received higher doses.

**Conclusion:**

Caffeine therapy in this unit showed marked variability in dosing, discontinuation and recommencement, highlighting the individualised nature of bedside decision-making, which may reflect clinical response to therapy.

**WHAT IS ALREADY KNOWN ON THIS TOPIC**

- Apnoea of prematurity can cause significant complications. Caffeine is the standard, effective and safe pharmacologic treatment.

**WHAT THIS STUDY ADDS**

- This study provides a picture of real-world caffeine prescribing in preterm infants, compared with a prescription guideline.
- Infants born at lower gestational ages received higher doses and discontinued caffeine at older postmenstrual ages.

**HOW THIS STUDY MIGHT AFFECT RESEARCH, PRACTICE OR POLICY**

- Non-invasive biomarkers are needed to support bedside decisions on caffeine initiation, adjustment and discontinuation.

## Introduction

 Apnoea of prematurity (AOP) commonly affects infants born before 32 weeks of gestational age (GA), with lower GA associated with recurrent AOP episodes.^1 2^ It is characterised by cessation of breathing due to immature respiratory control. This may result in bradycardia and desaturations and can contribute to adverse short-term and long-term outcomes including neurodevelopmental impairment.^3^,^6^ Caffeine is the mainstay of pharmacological management of AOP, widely used in neonatal care due to its broad therapeutic index and established efficacy.^7 8^

Despite its widespread use, caffeine administration is complex. While guidelines provide a framework for its administration,^9^ clinical practice often requires reviews based on the infant’s evolving clinical condition, including response to treatment, respiratory support requirements and the need for adjunct doxapram therapy. Caffeine dose may be reviewed multiple times during a preterm admission^10 11^; however, there is limited evidence on how caffeine therapy is modified over the course of a preterm admission. This study evaluates how caffeine therapy is modified throughout preterm admissions in a tertiary neonatal unit. It aims to describe patterns of dose adjustments, caffeine discontinuation and recommencement and evaluate associations with GA and respiratory support.

## Methods

### Study design and setting

This retrospective study was conducted at the John Radcliffe Hospital (JR Hospital), Oxford University Hospitals NHS Foundation Trust (OUH NHS Trust), UK. OUH NHS Trust guidelines^12 13^ recommend initiating caffeine citrate within the first hour of life for infants born before 32 weeks gestation, starting with 20 mg/kg intravenous loading dose followed 24 hours later by 5 mg/kg/day maintenance dose. Maintenance dosing may be increased daily by 5 mg/kg up to a maximum of 20 mg/kg/day, typically split into 10 mg/kg two times per day; with higher doses given at consultant discretion. The guideline states that caffeine is to be discontinued at 32–34 weeks postmenstrual age (PMA) or continued to 36 weeks if apnoea, bradycardia or respiratory support persists. In June 2024, the guideline was updated to recommend doxapram as a second-line agent.^12^ Only 19 infants received doxapram in our study; as the doxapram recommendation postdated the audited period this likely explains the relatively small number of infants that received doxapram and limits the robustness of any inferences. Doxapram-related content is therefore presented in online supplemental material.

### Population and inclusion criteria

This study focused on preterm infants born at or before 32 weeks of gestation admitted to the neonatal unit between 1 February 2022 and 31 October 2023, who received caffeine citrate for AOP. Infants were excluded if they did not receive caffeine. There were 311 admissions that met the criteria, of which 168 (54.0%) admission paper records were available for retrieval and review. Five admissions involved infants who were admitted a second time and received caffeine during both admissions, that is, records from 163 individual infants were included in the study. Records from other hospitals (ie, before/after transfer) were not included.

### Data collection

Eligible infants were identified through Badgernet electronic records and data were manually extracted from paper records. Data collected included caffeine start and stop dates, dose changes and documented reasons, route of administration, frequency and recommencement, alongside clinical variables such as GA, documented apnoeas, respiratory support and adjunct doxapram therapy to assess how caffeine therapy was modified in response to evolving clinical needs. Target doses (in mg/kg) were defined as the doses (in mg) that the clinicians intended to prescribe for every 1 kg of the infant’s body weight. Stat doses were single, one-off doses administered immediately, given in addition to the infant’s daily dose. Respiratory support included all oxygen therapies such as mechanical ventilation, high-flow and low-flow therapies, continuous positive airway ventilation, dual positive airway pressure and nasal intermittent positive pressure ventilation.

### Data analysis

The data were entered into a secure spreadsheet and analysed using MATLAB software. Continuous data were summarised with medians and IQR due to skewed distributions. Infants were grouped by GA in 1-week intervals to assess dosing patterns. Daily caffeine doses were extrapolated using the forward fill imputation method,^14^ which carries the most recently prescribed dose forward until a new dose was prescribed. Discontinuation of therapy was assumed for infants who were discharged (home) or if no doses were administered for at least 7 consecutive days prior to transfer or death. Regression analysis was used to evaluate associations between caffeine dosing and clinical factors including GA and respiratory support, with 95% CIs.

## Results

### Caffeine initiation

Infant demographics are provided in table 1. Of the 163 infants included, 131 (80.4%) were delivered at the JR and had records documenting the loading dose of caffeine. All received a loading dose of 20 mg/kg, with one receiving an additional single dose of 5 mg/kg (hence, a cumulative dose of 25 mg/kg) of caffeine on the first day of life. The median time of caffeine initiation was 1.52 (IQR: 1.22–1.98) hours after birth. Following loading, maintenance doses were mostly initiated at 5 mg/kg/day in 115 (87.8%) infants (table 2). All 131 infants started on intravenous caffeine before switching to oral. Thirty-one (18.5%) infants had a distinct documented switch from intravenous to oral at a median PMA of 31.9 (IQR: 28.6–32.3) weeks (table 2).

**Table 1:** Demographic characteristics

Characteristics	Value
Sex*	
Male; n (%)	101 (62.0)
Female; n (%)	62 (38.0)
Mode of delivery*	
Elective caesarean section; n (%)	15 (9.2)
Emergency caesarean section; n (%)	94 (57.7)
Forceps-assisted vaginal delivery; n (%)	3 (1.8)
Spontaneous vaginal delivery; n (%)	51 (31.3)
GA (weeks)	Range: 22.9–32.9
	Median (IQR): 28.3 (26.4–30.6)
Birth weight (kg)	Range: 0.390–2.185
	Median (IQR): 1.045 (0.773–1.400)
Duration of admission (days)	Range: 1–241
	Median (IQR): 36 (13 – 63)
Outcome of admission†	
Discharged; n (%)	64 (38.1)
Transferred to another hospital; n (%)	92 (54.8)
Died; n (%)	12 (7.1)
PMA at exit from the neonatal unit, JR Hospital	
PMA at discharge (weeks)	Range: 34.0–45.6
	Median (IQR): 37.0 (36.0–39.0)
PMA at transfer (weeks)	Range: 26.9–65.3
	Median (IQR): 32.0 (30.3–34.1)
PMA of babies that died (weeks)	Range: 23.1–38.7
	Median (IQR): 27.3 (24.7–30.9)

There were 163 infants with 168 admissions—5 infants were admitted twice.

* Denominator 163 infants.

† Denominator 168 admissions; n: frequency.

GA, gestational age; JR, John Radcliffe; PMA, postmenstrual age.

**Table 2:** Summary of caffeine administration

Characteristics	Value
Age at caffeine initiation (hours)*	Median (IQR): 1.52 (1.22–1.98)
First maintenance dose†	
5 mg/kg/day; n (%)	115 (87.8)
10 mg/kg/day; n (%)	5 (3.8)
5 mg/kg 12 hourly; n (%)	8 (6.1)
10 mg/kg 12 hourly; n (%)	1 (0.8)
Dose adjustments	
Number per admission	Median (IQR): 2 (1–3)
Total number of increases and decreases	213
Daily increases‡	
by 5 mg/kg per day; n (%)	93 (43.7)
by 10 mg/kg per day; n (%)	46 (21.6)
others; n (%)	7 (3.3)
Daily reductions‡	
by 5 mg/kg per day; n (%)	39 (18.3)
by 10 mg/kg per day; n (%)	25 (11.7)
others; n (%)	3 (1.4)
Switch from intravenous to oral§	
PMA (weeks)	Median (IQR): 31.9 (28.6–32.3)
Postnatal age (days)	Median (IQR): 6 (3–10)
Weight (kg)	Median (IQR): 1.315 (0.950–1.470)
Feed volume (mL/kg/day)	Median (IQR): 54 (24–96)
PMA at caffeine discontinuation (weeks)¶	Median (IQR): 34.0 (33.9–34.7)
Duration of respiratory support (days)**	Median (IQR): 21 (6–45)

* 131 infants.

† Denominator 129 infants.

‡ Denominator 213 adjustments.

§ 31 admissions.

¶ 81 admissions.

** 156 admissions.

n, frequency; PMA, postmenstrual age.

### Caffeine doses and adjustments

**Figure 1:**
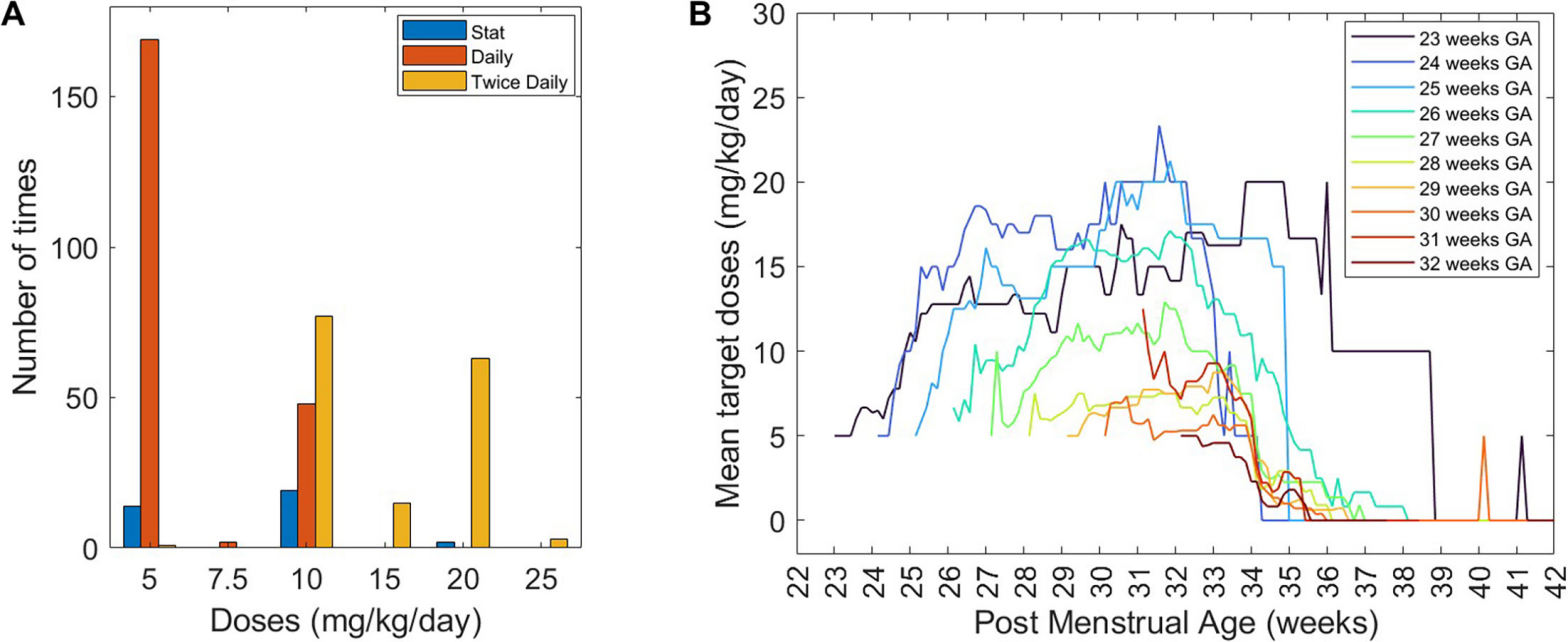
Maintenance target doses. (**A**) Prescribed daily maintenance doses and (**B**) mean target doses by postmenstrual age (PMA), grouped according to gestational age (GA) at birth. There were 12 infants in the 23 weeks GA group; this included two infants delivered at 22.9 weeks GA. Only two discontinued caffeine before exiting the neonatal unit (other infants died or were transferred before discontinuing caffeine); one discontinued at PMA 36.0 weeks, the other received the last dose of caffeine at 41.1 weeks giving rise to the angular nature of the plot at older PMA for this group.

The daily caffeine doses prescribed ranged from 5 mg/kg/day to 25 mg/kg/day as daily or two times per day (divided) doses. Higher doses were usually administered in two divided doses (figure 1A). In general, mean caffeine doses increased over the first 3 weeks (figure 1B). Babies born at a lower GA on average received caffeine for longer (stopping at older PMAs) and at higher doses compared with those born at older GAs (figure 1B).

**Figure 2:**
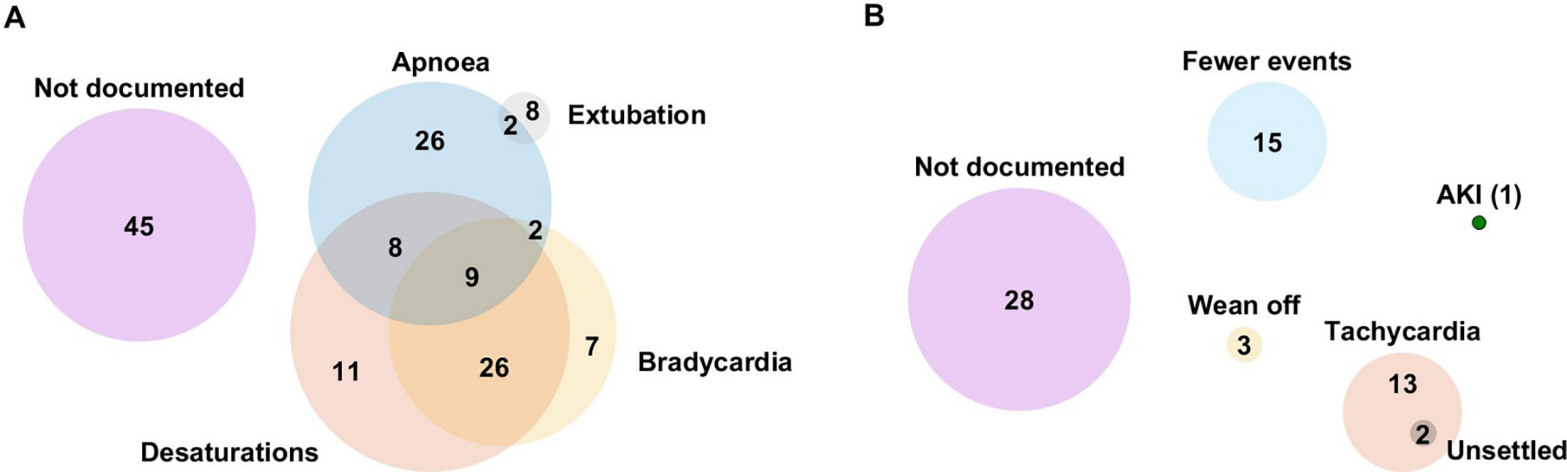
Reasons for caffeine dose changes. Documented reasons (extracted from the paper notes) for (**A**) increasing and (**B**) reducing caffeine doses. Fewer events: refer to fewer apnoeas, bradycardias and desaturations; number of admissions displayed in the diagrams. AKI, acute kidney injury.

On average, there were 2 (1–3) (median (IQR)) dose changes per admission with a maximum of eight changes. Target dose changes were mostly made in increments of 5 mg/kg/day (93 times (43.7%)) and reductions of 5 mg/kg/day (39 times (18.3%)); table 2. Desaturations (54) and apnoeas (47) were the most commonly documented reasons for dose increases (figure 2A). For dose reductions, tachycardia (15) and fewer events of apnoeas, bradycardias and desaturations (15) were the most frequently documented reasons (figure 2B). Single (stat) doses were administered 35 times, independent of the daily maintenance doses; 17 of these were for apnoeas, including 6 apnoeas associated with planned or accidental extubation.

### Caffeine discontinuation

**Figure 3:**
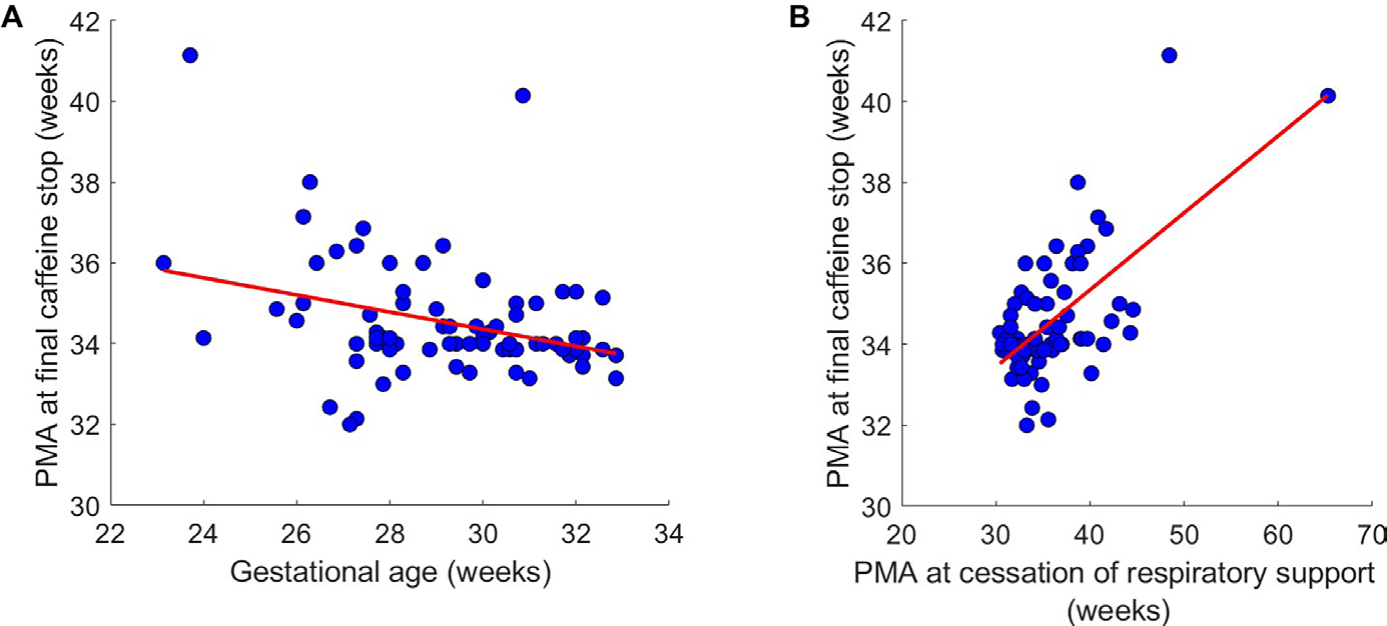
Cessation of caffeine. Postmenstrual age (PMA) at final caffeine stop compared with (**A**) gestational age and (**B**) PMA at cessation of respiratory support. Each dot indicates an admission, and the red line indicates the best line of fit.

Caffeine therapy was discontinued in 81 admissions at a median PMA of 34.0 (IQR: 33.9–34.7) weeks (table 2). (Note that other infants were transferred/died before stopping caffeine.) The total duration of caffeine therapy ranged from 1 to 105 days. The PMA at caffeine cessation decreased with GA (r(CI)=–0.33 (–0.51 to 0.12), p=0.0029; R²=0.11; figure 3A). The median interval between the last recorded apnoeic event and the final cessation of caffeine therapy was 14 (IQR: 3–28) days; however, seven admissions had recorded apnoeas after caffeine cessation. Caffeine therapy was recommenced in 4 of the 81 admissions (4.9%) after initial discontinuation. These babies differed in GA, PMA at caffeine cessation and duration of caffeine therapy (online supplemental figure 1). Reasons for recommencement included apnoeas (1), frequent desaturations and bradycardia (2) and desaturation only (1).

### Caffeine therapy and respiratory support

PMA at caffeine cessation correlated positively with both the duration of respiratory support (r(CI) 0.60 (0.44 to 0.73), p<0.0001; R²=0.36; online supplemental figure 2A) and PMA at the end of respiratory support (r(CI) 0.66 (0.50 to 0.77), p<0.0001; R²=0.43; figure 3B). Two infants requiring prolonged respiratory support beyond 45 weeks PMA received caffeine beyond 40 weeks PMA and were subsequently transferred to the paediatric intensive care unit.

There were 85 admissions (51.5%) involving mechanical ventilation, with a median duration of 2 (IQR: 1–9) days. Caffeine was discontinued in 30 of these admissions before the infants exited the neonatal unit at the JR (John Radcliffe Hospital). PMA at caffeine cessation correlated positively with PMA at extubation (r(CI) 0.68 (0.42 to 0.84), p<0.00001; R²=0.46; online supplemental figure 2B) and with the duration of mechanical ventilation (r(CI) 0.66 (0.52 to 0.77), p=0.0001; R² (CI) 0.44; online supplemental figure 2C).

## Discussion

This study highlights variability in daily caffeine citrate doses, target dose changes and patterns of discontinuation and recommencement. Despite its retrospective design and modest sample size, the findings contribute to our understanding of how clinical decisions are influenced by both guidelines and individual patient responses.

### Caffeine doses and adjustments

Daily caffeine citrate doses ranged from 5 to 25 mg/kg/day, reflecting standard maintenance dosing and adjustments based on clinical need. Caffeine is typically administered once per day, consistent with its prolonged half-life in neonates,^15^ although higher doses were divided into two times per day administrations. Evidence supporting two times per day dosing is limited. Faramarzi *et al*^16^ reported improved oxygen saturation with 2.5 mg/kg/dose two times per day compared with 5 mg/kg/dose/day. In contrast, Rebentisch *et al*^17^ observed no significant reduction in apnoea or bradycardia after switching preterm infants with intractable apnoeas from one time per day maintenance dose of 10 mg/kg to two times per day of 5 mg/kg; although tachycardia increased significantly with two times per day dosing.^17^

Infants in this study received additional single caffeine doses for apnoeas, comparable to the ‘mini-loads’ described by Storm *et al*,^11^ which did not abolish escalation to doxapram or mechanical ventilation, nor mortality rates.^11^ The increasing mean caffeine doses observed within the first 3 weeks of admission in this study are consistent with the previous study^11^ and coincide with periods of increased breathing pauses and declining caffeine concentrations reported in other studies.^18 19^ Although scheduled dose increases have been recommended to maintain therapeutic levels,^20 21^ and may be the rationale for the pattern observed in this study, routine dose increases will not be appropriate for all infants, given interindividual variability including genetic factors.^22^

Dose reductions were most often prompted by fewer episodes of clinical events, particularly apnoeas and desaturations. Tachycardia was another factor frequently cited, as a measurable sign of caffeine-related toxicity. Other potential adverse effects were not explicitly attributed to caffeine dose adjustments, while caffeine has well-described multiorgan and dose–response effects.^23^ This underlines the need for biomarkers to enhance therapeutic monitoring, especially in the absence of routine serum caffeine level monitoring. For example, electroencephalography-derived brain age has a significant relationship with apnoea rate,^24^ hence a combination of EEG-derived brain age and continuous vital signs monitoring using machine learning may better capture treatment need.^9^

### Caffeine discontinuation and recommencement

Caffeine was discontinued at a median PMA of 34 weeks. Discontinuation occurred at earlier PMA with higher GA, aligning with existing evidence of reduced risk of apnoea with age.^2^ The relatively long interval between the last recorded apnoea and caffeine cessation likely reflects continuation of therapy until the recommended PMA. However, seven infants had apnoeas after caffeine cessation, highlighting the need to consider other causes and potential biomarkers to guide optimal caffeine discontinuation. Similarly, a small number of infants resumed caffeine therapy, most commonly due to apnoeas, desaturations or bradycardia. The proportion of infants who restarted caffeine therapy in this study is lower than the 10% reported by Haddad *et al*,^25^ despite the similar PMA at discontinuation. Their higher incidence may reflect predefined criteria for reinitiation, including recurrent apnoeas requiring stimulation and/or abnormal sleep studies indicating central apnoea,^25^ which are not specified in the OUH NHS Trust guidelines.

### Caffeine therapy and respiratory support

Infants requiring prolonged respiratory support received longer caffeine therapy, with two infants receiving caffeine beyond 40 weeks PMA. Although caffeine has previously been linked with early extubation from mechanical ventilation and improved lung function of infants,^26^,^28^ infants with prolonged respiratory support needs, may require extended treatment. However, the benefit of extended caffeine therapy beyond term remains unclear. The MoCHA clinical trial reported no reduction in hospital stay and found increased rates of tachycardia and lower weight gain in the extended caffeine group.^29^ We did not explore the relationship between caffeine therapy and admission duration or outcomes such as mortality, as we did not obtain data on other factors known to influence discharge from neonatal unit such as comorbidities (eg, infection, surgical conditions) and the ability to establish full enteral feeding or maintain thermoregulation.^29^,^32^

## Conclusion

This study demonstrates that although caffeine guidelines provide a useful framework, significant individual variability exists in clinical practice. Dose titrations and therapy duration are often influenced by nuanced bedside assessments that reflect both measurable parameters like apnoeas and less well-defined signs of toxicity or recovery. There is a compelling need for further research into the development of non-invasive biomarkers or clinical decision-support tools that can aid in real-time adjustment of caffeine therapy.

## Supplementary material

10.1136/bmjpo-2025-004301 online supplemental file 1

## Data Availability

Data are available upon reasonable request.
