# Supplementary material for: Dosing practices of caffeine therapy for apnoea of prematurity: a retrospective single-centre observational study
Source: BMJ Paediatr Open. 2026 Mar 3;10(1):e004301. doi: 10.1136/bmjpo-2025-004301 (PMC12959035; doi:10.1136/bmjpo-2025-004301)
Supplement: online supplemental file 1 [file bmjpo-10-1-s001.docx]

**Supplementary material: DOSING PRACTICES OF CAFFEINE THERAPY FOR APNOEA OF PREMATURITY: A RETROSPECTIVE SINGLE-CENTRE OBSERVATIONAL STUDY**

**AUTHORS:**

Odunayo A. T. Fatunla, Coen S. Zandvoort, Shellie Robinson, Eleri Adams, Caroline Hartley

**Caffeine and Doxapram Therapy**

Nineteen admissions (11.3%) involved the administration of doxapram at a dose of 2mg/kg/hour, initiated at a PMA of 27.4 (IQR:26.7–30.1) weeks. At doxapram initiation, the median concurrent caffeine dose was 15 (IQR:7.5–20) mg/kg/day. Following doxapram therapy initiation, caffeine doses were adjusted in 9 admissions: 6 doses were increased, 2 doses were reduced, and 1 dose was temporarily discontinued for 2 days. After doxapram cessation, caffeine doses were increased in 3 admissions and reduced in 3 others, no caffeine dose changes were made in the others.

The role of doxapram in preventing apnoea is not well defined, but it has shown effectiveness as an alternative or adjunct therapy to caffeine in preventing reintubation and treating refractory apnoeas [1,2]. Doxapram was used in a small minority of infants in this study, therefore, no conclusions regarding the required caffeine adjustments can be drawn. However, given that doxapram shares some side effects with caffeine such as tachycardia, close monitoring is necessary during co-administration [1–4]. Ongoing studies such as the DOXA-trial [5] may help to clarify its role as a second-line therapy, and any required caffeine dose adjustments, and inform more precise guidelines for its use in the management of AOP.

**References**

1. Evans S, Avdic E, Pessano S, Fiander M, Soll R, Bruschettini M. Doxapram for the prevention and treatment of apnea in preterm infants. Cochrane Database of Systematic Reviews [Internet]. 2023 [cited 2023 Dec 5];(10). Available from: https://www.cochranelibrary.com/cdsr/doi/10.1002/14651858.CD014145.pub2/references

2. Poppe JA, van Weteringen W, Sebek LLG, Knibbe CAJ, Reiss IKM, Simons SHP, et al. Precision Dosing of Doxapram in Preterm Infants Using Continuous Pharmacodynamic Data and Model-Based Pharmacokinetics: An Illustrative Case Series. Frontiers in Pharmacology [Internet]. 2020 [cited 2022 Aug 10];11. Available from: https://www.frontiersin.org/articles/10.3389/fphar.2020.00665

3. Ainsworth L, Adams E. Apnoea of prematurity: Caffeine and Doxapram. Newborn Care Services Guidelines. 2024.

4. Abdel-Hady H, Nasef N, Shabaan AE, Nour I. Caffeine therapy in preterm infants. World J Clin Pediatr. 2015 Nov 8;4(4):81–93.

5. Poppe JA, Flint RB, Smits A, Willemsen SP, Storm KK, Nuytemans DH, et al. Doxapram versus placebo in preterm newborns: a study protocol for an international double blinded multicentre randomized controlled trial (DOXA-trial). Trials. 2023 Oct 10;24(1):656.

**Supplementary Figure 1: Infants that restarted caffeine**

Line plots of the course of caffeine therapy during admission for the four infants that restarted caffeine

**Supplementary Figure 2: Caffeine cessation and respiratory support**

(A). Total respiratory support duration and postmenstrual age (PMA) at final caffeine stop (B). PMAs at extubation and final caffeine stop and (C). Total mechanical ventilation duration and PMA at final caffeine stop

Each blue dot indicates an admission, and the red line indicates the best line of fit
