# Supplementary material for: Dosing practices of caffeine therapy for apnoea of prematurity: a retrospective single-centre observational study
Source: BMJ Paediatr Open. 2026 Mar 3;10(1):e004301. doi: 10.1136/bmjpo-2025-004301 (PMC12959035; doi:10.1136/bmjpo-2025-004301)
Supplement: Supplementary file 2 [file bmjpo-2025-004301.reviewer_comments.pdf]

PEER REVIEW HISTORY

BMJ Paediatrics Open publishes all reviews undertaken for accepted manuscripts. Reviewers are asked to complete a checklist review form and are provided with free text boxes to elaborate on their assessment. These free text comments are reproduced below.

ARTICLE DETAILS

**TITLE (PROVISIONAL)**	DOSING PRACTICES OF CAFFEINE THERAPY FOR APNOEA OF PREMATURITY: A RETROSPECTIVE SINGLE-CENTRE OBSERVATIONAL STUDY AUTHORS
**AUTHORS**	Fatunla, Odunayo Adebukola Temitope<br>Zandvoort, Coen S<br>Robinson, Shellie<br>Adams, Eleri<br>Hartley, Caroline

VERSION 1 – REVIEW

**REVIEWER NAME**	Pournami, Femitha
**REVIEWER AFFILIATION**	
**REVIEWER CONFLICT OF INTEREST**	No Conflict of Interest
**AI DISCLOSURE**	No, I have not used AI
**DATE REVIEW RETURNED**	23-Dec-2025

**GENERAL COMMENTS**	Thank your for addressing an important area of research.<br>I did not find in the manuscript, details of what protocol the unit uses for up gradation of doses, and more importantly for stopping therapy<br>There are publications to support use of caffeine for up to 34 weeks or for 1 week apnea free period; whichever is longer<br>The only part of caffeine therapy protocol clearly defined in your manuscript is for whom to start therapy; but not the other components<br>It is therefore quite obvious that there will indeed be several variations in practice<br>It might have been much better and more useful to readers if the protocol was outlined and then compliance to this protocol is audited and measured.<br>Here, in this manuscript in its form just now is only a pure description of real world demonstration of variation in practice that seems far too individualised for current day evidence based medicine.<br><br>there is no primary measurable outcome defined up front, and therefore no sample size calculation, and no derivable conclusions in the real sense.<br><br>I would suggest that the text be markedly shortened to fit a short correspondence or scientific letter; whichever suits the journal’s requirements.<br>The findings are not novel and may not be wholly suitable to be published as a full fledged original research paper.

**REVIEWER NAME**	Shaniv, Dotan

**REVIEWER AFFILIATION**	Kaplan Medical Center, Pharmacy Services
**REVIEWER CONFLICT OF INTEREST**	No Conflict of Interest
**AI DISCLOSURE**	No, I have not used AI
**DATE REVIEW RETURNED**	27-Dec-2025

**GENERAL COMMENTS**	Dear editor,<br><br>Thank you for the opportunity to review the manuscript titled: "Dosing Practices of Caffeine Therapy for Apnoea of Prematurity: A Retrospective Single-Centre Observational Study". This study provides a glimpse at dosing practices of caffeine in a certain newborn care unit in the UK, which may be useful as a reference for other units to compare practices, as well as a self-check of practices to improve neonatal pharmacotherapy of one of the most common pharmacological treatments in the NICU.<br>As this report covers many aspects of caffeine treatment, its descriptive nature suggests it might be useful to try and condense it some more, to allow for key points to "shine". In the Results section, perhaps consider converting certain subsections into tables for better clarity and ease of assessment by readers? Most subsections could be considered for this, particularly "Caffeine initiation" and "Discontinuation".<br>The Discussion section could also be made shorter; structuring it with subsections may help focusing on the main findings and their interpretation.<br>Additional, more specific points to consider:<br>1. The authors mention and cite the OUH NHS Trust guidelines, but I was unable to find and access them. Are they freely accessible? If not, perhaps cite the relevant part that deals with dosing recommendations for caffeine?<br>2. The discussion on twice daily dosing of caffeine should include a reference to its very long half life in newborns, which is why there is no point in such a regimen. The authors may use the 2020 Aranda and Beharry paper as a reference: https://pubmed.ncbi.nlm.nih.gov/33293242/<br>3. In the Demographic characteristics subsection of the Results, the second sentence should be structured consistently regarding numbers and percentages.<br>4. In Table 1, is the statistic column truly necessary? Perhaps it is possible to integrate it into the Characteristics column to reduce "clutter" in the table?<br>5. Whenever reporting regression results, the authors should consider reporting not only R-squared, but also the Pearson coefficient (r), to indicate the strength and the direction of the linear association.<br>6. Where switching from IV to oral caffeine is discussed, were there newborns switched back from oral to IV, due to NEC, feeding intolerance, etc.? Might be interesting to include this information.<br>7. Under "Caffeine Doses and Adjustments", it may be unnecessary to specifically mention that 5 mg/kg/day in two divided doses was prescribed only once?<br>8. Authors should explain what "Single (one-off) doses" are.<br>9. It is mentioned that the median interval between the last recorded apnoeic event and the final cessation of caffeine therapy was 15 days. Seems rather long? Was an explanation suggested in the Discussion section? I do not recall seeing one.<br>10. This might be just a technical hiccup, but under "Caffeine therapy and respiratory support" the figures in the supplementary

	material are referenced, and it seems that figures 1C and 1D have accidentally been switched?<br>11. Also, in the review PDF, the figures in the end do not appear in the order of the figure legends that precede them (figures 1 and 3 were switched).

VERSION 1 – AUTHOR RESPONSE

Reviewer: 1
Femitha Pournami
Comments to the Author
Thank you for addressing an important area of research.
I did not find in the manuscript, details of what protocol the unit uses for up gradation of doses, and more importantly for stopping therapy
There are publications to support use of caffeine for up to 34 weeks or for 1 week apnea free period; whichever is longer
The only part of caffeine therapy protocol clearly defined in your manuscript is for whom to start therapy; but not the other components
It is therefore quite obvious that there will indeed be several variations in practice
It might have been much better and more useful to readers if the protocol was outlined and then compliance to this protocol is audited and measured.
Here, in this manuscript in its form just now is only a pure description of real world demonstration of variation in practice that seems far too individualised for current day evidence based medicine.
Thank you for your comments. The details of the unit protocol have been summarised in the methods section, and the information on stopping therapy has now been included. This reads “Caffeine is to be discontinued at 32–34 weeks PMA, or continued to 36 weeks if apnoea, bradycardia or respiratory support persists”.
We agree that this study is limited in that it is conducted at a single centre. Nevertheless, it highlights the gap between recommendations and clinical practice, and emphasising the need for validated bedside biomarkers to guide dose escalation and cessation, and to improve individualised caffeine therapy in neonatal care.

there is no primary measurable outcome defined up front, and therefore no sample size calculation, and no derivable conclusions in the real sense.
Thank you for your observation. The study was descriptive rather than to test a predefined primary outcome or hypothesis, therefore, there was no sample size calculation. The study was designed to include all preterm infants born at <32 weeks gestational age and admitted between February 2022 and October 2023, who received caffeine.

I would suggest that the text be markedly shortened to fit a short correspondence or scientific letter; whichever suits the journal’s requirements.
The findings are not novel and may not be wholly suitable to be published as a full fledged original research paper.

Thank you for your comments. We have shortened the manuscript substantially as advised.

Reviewer: 2
Mr. Dotan Shaniv, Kaplan Medical Center
Comments to the Author
Dear editor,

Thank you for the opportunity to review the manuscript titled: “Dosing Practices of Caffeine Therapy

for Apnoea of Prematurity: A Retrospective Single-Centre Observational Study”. This study provides a glimpse at dosing practices of caffeine in a certain newborn care unit in the UK, which may be useful as a reference for other units to compare practices, as well as a self-check of practices to improve neonatal pharmacotherapy of one of the most common pharmacological treatments in the NICU.
As this report covers many aspects of caffeine treatment, its descriptive nature suggests it might be useful to try and condense it some more, to allow for key points to “shine”.
Thank you very much for your comments. We have condensed the manuscript, highlighting the key points of the study
In the Results section, perhaps consider converting certain subsections into tables for better clarity and ease of assessment by readers? Most subsections could be considered for this, particularly “Caffeine initiation” and “Discontinuation”.
Thank you for your suggestions. We have now created Table 2: Summary of caffeine administration to contain the descriptive statistics and enable easier reading.

The Discussion section could also be made shorter; structuring it with subsections may help focusing on the main findings and their interpretation.
The discussion section is now shorter and structured.

Additional, more specific points to consider:
1. The authors mention and cite the OUH NHS Trust guidelines, but I was unable to find and access them. Are they freely accessible? If not, perhaps cite the relevant part that deals with dosing recommendations for caffeine?
The guidelines are not freely accessible but we have summarised the protocol in the methods section and edited it to include the previously omitted part on the discontinuation of caffeine. We have now included this sentence: “Caffeine is to be discontinued at 32–34 weeks PMA, or continued to 36 weeks if apnoea, bradycardia or respiratory support persists”.

2. The discussion on twice daily dosing of caffeine should include a reference to its very long half life in newborns, which is why there is no point in such a regimen. The authors may use the 2020 Aranda and Beharry paper as a reference: https://pubmed.ncbi.nlm.nih.gov/33293242/
Thank you for your suggestion, we have incorporated this into the discussion section, which now reads: “Caffeine is typically administered once daily, consistent with its prolonged half-life in neonates [15]”.

3. In the Demographic characteristics subsection of the Results, the second sentence should be structured consistently regarding numbers and percentages.
We have revised the manuscript to maintain consistency in describing the demographic characteristics.

4. In Table 1, is the statistic column truly necessary? Perhaps it is possible to integrate it into the Characteristics column to reduce “clutter” in the table?
Thank you for your observation. The Tables have now been formatted as suggested.

5. Whenever reporting regression results, the authors should consider reporting not only R-squared, but also the Pearson coefficient (r), to indicate the strength and the direction of the linear association.
The Pearson coefficient (r) and the respective confidence intervals have now been incorporated.

6. Where switching from IV to oral caffeine is discussed, were there newborns switched back from oral to IV, due to NEC, feeding intolerance, etc.? Might be interesting to include this information.
Thank you for this helpful feedback. Unfortunately, we did not retrieve this information from the paper notes, and we suggest that it should be collected in future studies.

7. Under “Caffeine Doses and Adjustments”, it may be unnecessary to specifically mention that 5 mg/kg/day in two divided doses was prescribed only once?
We have removed this from the manuscript as suggested.

8. Authors should explain what “Single (one-off) doses” are.
We have incorporated this into the manuscript. The data collection subsection of the manuscript now includes “Stat doses were single, one-off doses administered immediately, given in addition to the infant’s daily dose.”

9. It is mentioned that the median interval between the last recorded apnoeic event and the final cessation of caffeine therapy was 15 days. Seems rather long? Was an explanation suggested in the Discussion section? I do not recall seeing one.
Thank you for your observation. This has now been included: “The relatively long interval between the last recorded apnoea and caffeine cessation likely reflects continuation of therapy until the recommended PMA.”

10. This might be just a technical hiccup, but under “Caffeine therapy and respiratory support” the figures in the supplementary material are referenced, and it seems that figures 1C and 1D have accidentally been switched?
Thank you, we have now corrected this error.

11. Also, in the review PDF, the figures in the end do not appear in the order of the figure legends that precede them (figures 1 and 3 were switched).
Thank you, we have now corrected this error.
